# Canine companionship is associated with modification of attentional bias in posttraumatic stress disorder

**DOI:** 10.1371/journal.pone.0179912

**Published:** 2017-10-11

**Authors:** Steven H. Woodward, Andrea L. Jamison, Sasha Gala, Tyson H. Holmes

**Affiliations:** 1 National Center for PTSD, Dissemination and Training Division, VA Palo Alto Health Care System, Palo Alto, CA, United States of America; 2 Human Immune Monitoring Center (HIMC), Institute for Immunity, Transplantation and Infection, Stanford University School of Medicine, Stanford, CA, United States of America; University of Akron, Akron, Ohio, UNITED STATES

## Abstract

Attentional bias towards aversive stimuli has been demonstrated in the anxiety disorders and in posttraumatic stress disorder, and attentional bias modification has been proposed as a candidate treatment. This study rigorously assessed attentional bias towards aversive and pleasant visual imagery associated with the presence or absence of a familiar service canine in 23 veterans with chronic military-related posttraumatic stress disorder. Participants were repeatedly tested with and without their service canines present on two tasks designed to elicit spontaneous visual attention to facial and scenic image pairs, respectively. Each stimulus contrasted an emotive image with a neutral image. Via eye-tracking, the difference in visual attention directed to each image was analyzed as a function of the valence contrast and presence/absence of the canine. Across both tasks, the presence of a familiar service canine attenuated the normative attentional bias towards aversive image content. In the facial task, presence of the service canine specifically reduced attention toward angry faces. In that task, as well, accumulated days with the service canine similarly modulated attention toward facial emotion. The results suggest that the presence of a familiar service canine is associated with attenuation of attentional bias to aversive stimuli in chronic military-service-related posttraumatic stress disorder. Questions remain regarding the generalization of such effects to other populations, their dependence on the familiarity, breed, and training of the canine, and on social context.

## Significance

A wealth of anecdotal data supports the euthymic impact of canine companionship on some individuals; and this effect is strongly endorsed by many combat veterans diagnosed with posttraumatic stress disorder. We employed a within-subjects longitudinal design to provide a rigorous test of the impact of the presence of a service canine on attentional bias in a veteran sample. The presence of a familiar canine was associated with modification of attentional bias away from aversive and towards pleasant visual stimuli.

## Introduction

Abundant anecdotes, qualitative findings, and uncontrolled observational studies have supported the positive impact(s) of canine companionship in many populations, including military personnel with posttraumatic stress disorder and related conditions [PTSD; 1, 2], but rigorous research is lacking. Attentional bias towards threatening or aversive stimuli is a common feature of anxiety disorders [3, 4] and has been observed in PTSD [5]. Modification of attentional bias has emerged as a candidate treatment across a range of psychiatric diagnoses including PTSD [6–11]. The present study was designed to provide a rigorous test of the hypothesis that canine companionship modifies attentional bias in chronic severe military-service-related PTSD in a positive direction.

Attentional bias has been assessed using a variety of laboratory paradigms and yielded varied outcomes [3, 5]; however, studies directly quantifying attention using eye-tracking have consistently shown that persons with PTSD preferentially attend to threatening and to generically aversive stimuli [12–16]. We employed eye-tracking to assess whether the presence of a canine companion modified visual attention to two sets of stimuli, emotive scenes and emotive faces, in military veterans diagnosed with chronic severe PTSD. Though similar in structure, the two tasks differed in their reliance on the processing of social (facial affect) stimuli and so provided information as to whether an effect of canine companionship, if observed, was specific to that domain or, rather, was generic. This distinction is of interest because canine companionship appears to promote human-to-human sociality across a number of contexts [17–20], and PTSD is associated with impaired social function [21–29].

## Materials and methods

### Participants

The 23 participants in this study were male U.S. military veterans, primarily of the Iraq and Afghanistan wars, engaged in inpatient treatment in the Trauma Recovery Program (TRP) at the Veterans Affairs Palo Alto Health Care System. All participants provided written informed consent to undergo the described procedures which were performed under the aegis of the Stanford Administrative Panels on Human Subjects in Medical Research who specifically approved this study. (As the service canines underwent no experimental procedures, oversight by Institutional Animal Care and Use Committees was not required.)

All patients in the TRP received a mix of group-based cognitive-behavioral interventions delivered in the context of a therapeutic milieu. To individualize treatment, optional groups were also offered, among them a Service Animal Training Intervention (SATI) in which residents provided early socialization to young service canines provided by a non-profit organization (Paws for Purple Hearts) under the supervision of a professional trainer. The canines were exclusively Labrador and Golden Retrievers bred to achieve the highest levels of aid to mobility-impaired veterans. For the participants, the SATI provided opportunities to reinforce skills addressed by formal treatments, such as effective communication and behavioral activation. Patients applied to participate in the SATI subject to approval by TRP staff with no input from study staff. Criteria for acceptance into the SATI program included relative behavioral stability, no known fall risk, and consistent engagement with the therapeutic milieu. On admission to the SATI, participants joined a two-patient team that alternated custody of the canine. On “canine-plus” days, the custodial trainer remained with the canine at virtually all times, including during treatment groups, program outings, and at night. On “canine-minus” days, the non-custodial trainer had little contact with the canine. SATI participants did not work with their canines on weekends. SATI participation typically continued for six or more weeks unless participants discharged.

### Psychometrics

Psychiatric diagnoses were obtained via the Clinician Administered PTSD Scale for DSM-5 (CAPS-5) [30] and the Structured Clinical Interview for DSM-5 (SCID-5) [31]. Also administered were the Combat Exposure Scale (CES) [32], PTSD Checklist– 5 (PCL-5) [33], Beck Depression Inventory II (BDI) [34], Difficulty with Emotion Regulation Scale (DERS) [35], and Emotion Regulation Questionnaire (ERQ) [36].

### Procedures

During their participation in the SATI and for up to six weeks, participants came each Thursday or Friday to the laboratory located on the same campus as the TRP. Electrodes and sensors were applied enabling the collection of corrugator electromyogram, electrocardiogram, electrodermal responses, and respiratory movements. (Psychophysiological measures were collected as outcomes in loud tone and math stressor tasks that followed the attentional bias assessments and will be discussed in another report.) Participants were seated in a comfortable chair in the testing chamber, approximately 100 cm from a 58 cm (diagonal) computer monitor. The eye tracking apparatus (facelab^TM^, Seeing Machines, Canberra, Australia) required no equipment attached to participants and no head restraint. Calibration of eye tracking required five to ten minutes. When present, the service canine lay on the floor next to the participant.

Participants engaged in a one-hour protocol including a computer-delivered version of the Reading the Mind in the Eyes Test [37], followed by the two attention bias tasks reported, here. Written instructions were provided on-screen prior to each task. After testing, sensor detachment required approximately five minutes after which participants were debriefed. The order of the facial emotion and scene tasks was alternated over sessions within participants. Participants were administered a different equivalent form of each task at every session.

### Stimuli

To promote unstructured viewing, the attentional bias tasks used relatively long, seven-second, trial durations and low-demand cover tasks requiring only vocal responses. Both tasks were programmed in eprime^TM^ (Psychology Software Tools, Sharpsburg, PA) and each required approximately ten minutes to complete.

### Emotive scenes task

Six equivalent forms of an assessment of attentional bias to emotive scenes were developed using images from the International Affective Picture System (IAPS) [38]. A stimulus consisted of an emotive image paired with a neutral image. In each set, 16 aversive images and 16 pleasant images were paired with 32 neutral images, with each image pair presented in both left-right orientations for a total of 64 trials. Each image was 15 cm high by 21.5 cm wide and were 3.5 cm apart. Images with military and male homosexual content were excluded. The forms were balanced for mean valence and arousal of aversive and pleasant images and for mean valence difference across emotive and neutral images within stimuli (using ratings provided by male samples [38]). The order of emotive image valence was randomized. (Listings of the stimuli utilized in each task are available in Tables A and B in S1 File) Each trial began with a fixation number presented for 500 milliseconds (msec) which participants reported aloud. Vocalizations were recorded for later scoring. Fifteen hundred msec after the offset of the fixation number, the stimulus appeared for seven seconds. Participants were not further instructed. The inter-trial interval varied randomly from 500 to 1500 msec.

### Facial emotion task

Six equivalent forms of an assessment of attentional bias to facial emotion were derived from the NimStim images [39], a set of 646 color photographs of the faces of young actors expressing a range of emotions in a posture of direct gaze taken against a white background. The faces were 24 cm high by 21 cm wide and were 4 cm apart. Each set of trials included six angry, six fearful, and twelve happy facial expressions. Each emotive expression was paired with a calm/neutral expression provided by the same actor. (Though nominally separate categories in the NimStim corpus, calm and neutral were merged for the purposes of this study.) Each emotive/neutral pair was presented in both horizontal orientations for a total of 48 trials per set. Within each set of 48 trials, actor gender was balanced and the order of emotions randomized. Each of the forms utilized different actors. Each trial began with a fixation number presented for 500 milliseconds (msec) which participants reported aloud. After 1500 msec, a face pair was presented for seven seconds, followed by the question “Male or female?” presented in the center of the screen for one second. Participants then reported the gender of the actor. Vocalizations were recorded for later scoring. The inter-trial interval varied randomly from 2 to 4.5 seconds. The fixation number reporting cover tasks were employed to promote central fixation prior to stimulus onset. The gender reporting task was employed both to induce participants to scan both faces and to increase engagement with the faces tasks which was less intrinsically interesting than the scenes task.

### Processing of eye tracking data

The facelab^TM^ system tracked the intersection of the gaze vector and the image plane with better than one degree of visual angle precision at a sampling rate of 60 Hz. Signals output coincident with image onsets allowed for precise stimulus-response synchronization. Inspection time was estimated as the sum of gaze samples falling within an image boundary during each stimulus presentation. As the NimStim facial locations varied slightly, rectangles centered on the faces were measured on each stimulus and stored in a file that the eye-tracking quantification software accessed when calculating inspections times. (Example stimuli and gaze tracks are provided in Fig A in S1 File)

### Statistics

SATI participants attended between two and six laboratory sessions (mean = 5.5). All the data provided by the 23 participants (120 sessions) are included in this report, subject to the application of a single tracking data quality criterion according to which the sum of inspection times over the two members of an image pair was required to exceed 100 msec in order for the trial to be retained. Application of this criterion excluded trials contaminated by tracking failure and/or by inattention. Approximately 80% of all trials were retained, with lost trials resulting from some combination of inattention and/or the deleterious impact of light eye colors and/or glasses on gaze tracking. (Requiring up to 3000 msec of viewing time per trial had negligible impact on the results.) For both experiments, attentional bias was operationalized as the difference between total gaze duration directed to the emotive versus the neutral image, calculated per trial. This variable exhibited moderately leptokurtic distributions with little skew (See Fig B in S1 File). Using linear mixed effects modeling [40, 41] attentional bias was modeled principally as a function of the interaction of stimulus contrast, and either the presence/absence of the service canine in the testing session, or accumulated days with the service canine. While set could have been modeled as a random factor, preliminary analyses of the facial emotion data suggested that set was not independent of all fixed effects in that model; hence it was treated as a fixed effect throughout. Accumulated days with the service canine was reduced to a two-level factor via a median split, contrasting 0–8 days of companionship with 9–25 days of companionship. Presence/absence of service canine and accumulated days with dog were uncorrelated (phi = 0.07). Days from admission to the TRP to the first test session was entered as an among-subjects covariate. When analyzing scene data, the absolute value of the difference between the valences of the emotive and foil stimuli [per the IAPS manual; 38] was included as a trial-varying covariate. A random intercept was specified for each canine and for each participant to account for possible within-canine and within-participant clustering of observations and to adjust for otherwise unmeasured participant-level traits.

## Results

### Sample characteristics

Mean age of the sample was 39 years (s.d. = 13). Participants self-identified ethnicity from a list including White (67%); African-American, not Hispanic (11%); Hispanic, White (5%); Hispanic, African-American (0%); American-Indian/Alaskan (0%); Asian; Pacific Islander (11%), and Other (5%). The distribution of self-identified ethnicity was representative of the VA patient population. All participants met criterion for PTSD. Mean CAPS-5 total severity score was 40.4 (standard deviation = 9.4). Consistent with inpatient PTSD treatment status, 65% also met criteria for current Major Depressive Disorder (MDD) and 87% for lifetime MDD. 70% met criteria for current (past 12 months) Alcohol Use Disorder (AUD) and 96% for lifetime AUD. 35% met criteria for a current (past 12 months) Substance Use Disorder (SUD) and 61% for lifetime SUD. Medication use was as follows: specific serotinergic reuptake inhibitors or serotonin-norepinephrine reuptake inhibitors, 74%, anticonvulsant—mood stabilizers, 43%, alpha-1 noradrenergic antagonists, 26%, opioid antagonists, 22%, atypical anti-psychotics, 17%, trazodone, 17%, mirtazapine, 17%, opioids, 13%, anxiolytics, 13%, tricylic antidepressants, 4%, buproprion, 4%. Mean scores on self-report measures were as follows: CES, 18.5 (s.d. = 12.5), PCL-5, 54.6 (s.d. = 14.3), BDI, 27.9 (s.d. = 9.0), DERS, 111.2 (s.d. = 28.2), and ERQ, 40.5 (s.d. = 9.7; reappraisal = 21.6, s.d. = 7.7; suppression = 18.9, s.d. = 5.1). All of these results are consistent with chronic severe PTSD as seen in Veterans.

Whether or not the fixation number or model gender was correctly reported had negligible impact on the results and will not be discussed further.

### Emotive scenes task

**Fig 1:**
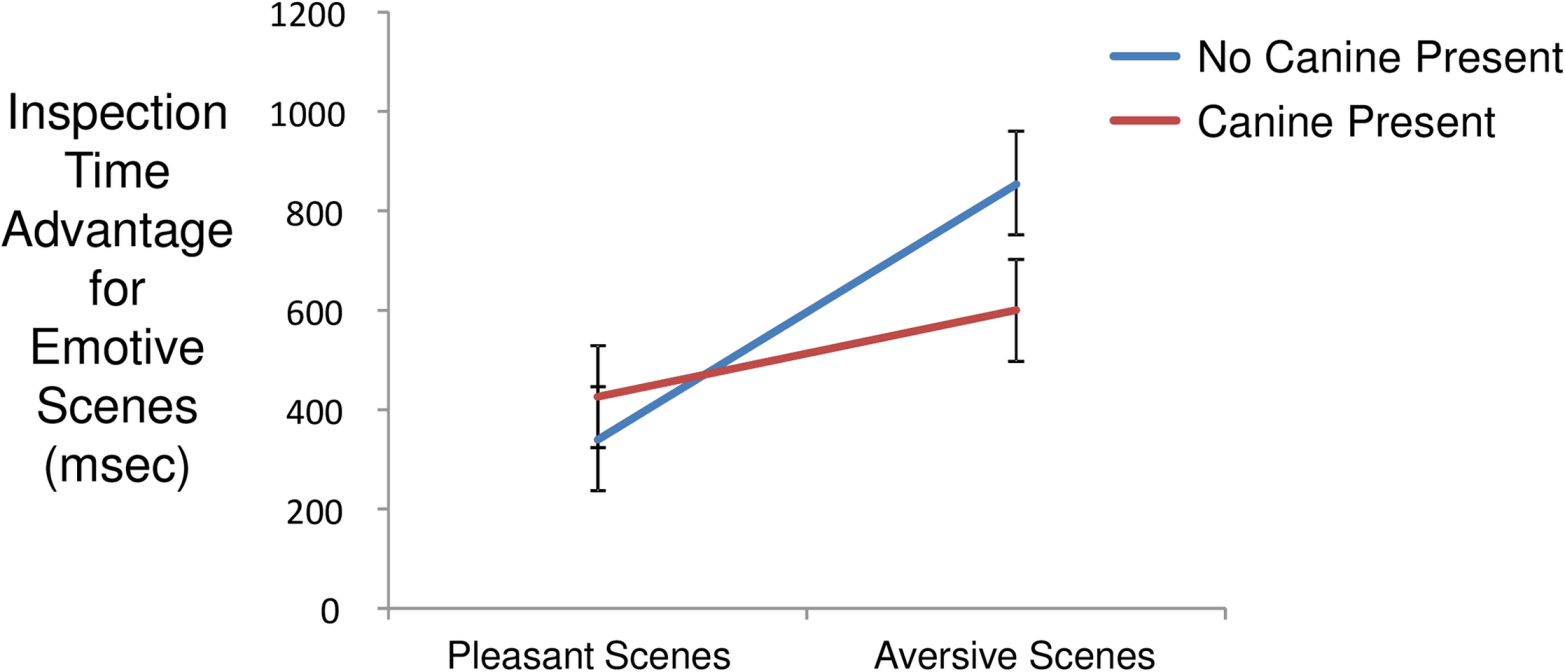
Interaction of contrast and canine presence on inspection time differences favoring emotive over neutral scenes. Population marginal means derived from fitted model. Error bars are standard errors.

Mean visual inspection time for paired scenic stimuli was 3140 msec (s.d. = 1814 msec). Within scene pairs, mean inspection time for emotive scenes was 1828 msec (s.d. = 1370 msec) and for neutral scenes was 1312 msec (s.d. = 1155 msec). Per-trial inspection time differences elicited by emotive versus neutral scenes exhibited a mean of 516 msec (s.d. = 1768 msec) in favor of the former, indicative of a general attentional bias in favor of emotive (both aversive and pleasant) over neutral stimuli. Plotted in Fig 1, modeling of inspection time differences detected a significant main effect of valence contrast (aversive > pleasant; F(1,3091) = 42.4, p < 0.0001) and an interaction of valence contrast with canine presence (F(1, 3091) = 11.6, p = 0.0007) such that canine presence attenuated the bias of visual attention towards aversive content. (Probability plots of model residuals are provided in Fig C in S1 File) The effect of a valence difference covariate based on IAPS norms [42] was significant (F(1,3091) = 5.69, p = 0.017) with larger normative valence differences associated with larger attentional biases. Days post admission to the TRP was not associated with an effect on attentional bias to emotive scenes (F(1,69) = 0.04, p = 0.84). Decomposing the interaction of valence contrast and canine presence, the reduction of bias towards aversive scenes was statistically significant (t(69) = 2.56, p = 0.013), while the increase of bias towards pleasant scenes was not (t(69) = 0.87, p = 0.39). The model including days accumulated with canine exhibited only a main effect of contrast (F(1,3092) = 47.9, p < 0.0001; days accumulated with canine: F(1,71) = 0.4, p = 0.55); contrast x days accumulated with canine: F(1,3092) = 1.42, p = 0.23).

### Facial emotion task

**Fig 2:**
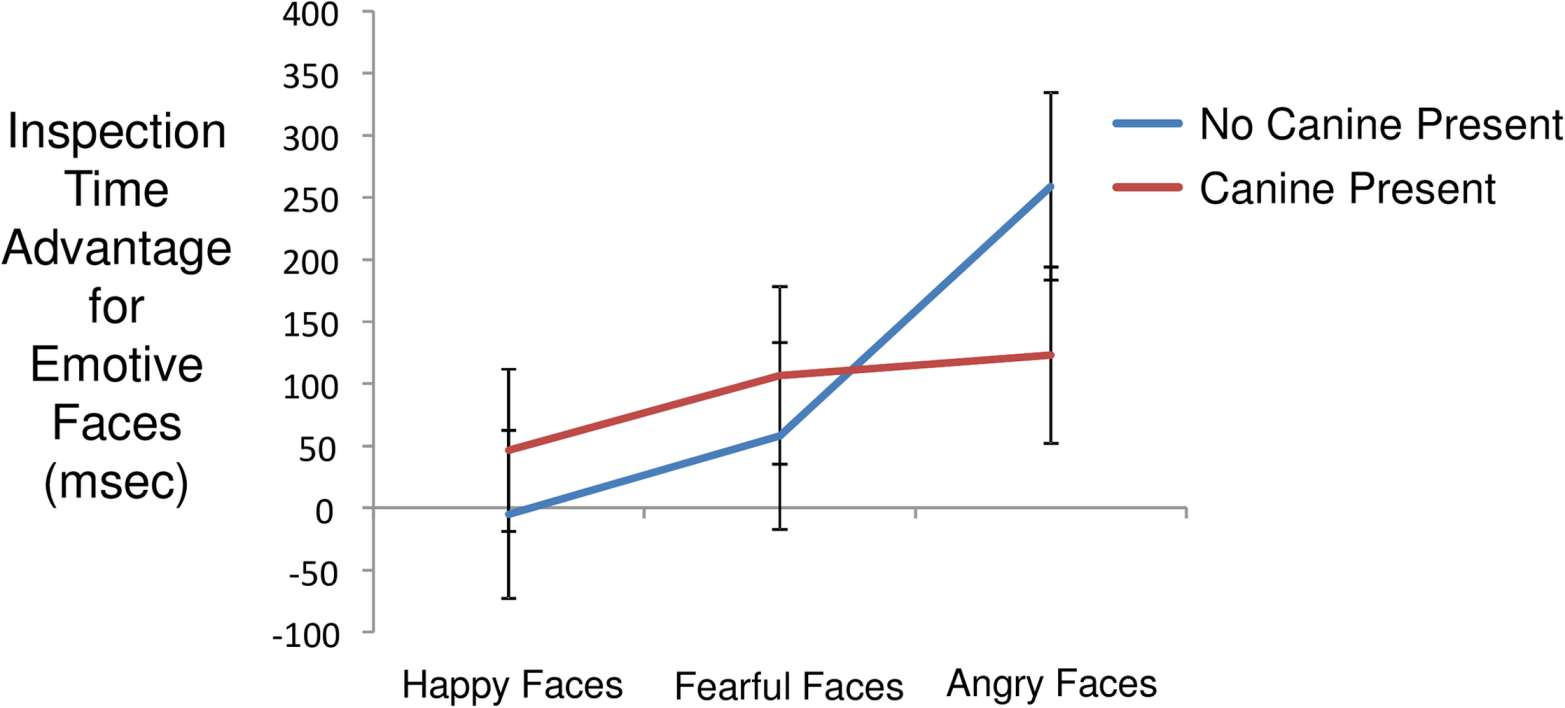
Interaction of contrast and canine presence on inspection time differences favoring emotive over neutral faces. Population marginal means derived from fitted model. Error bars are standard errors.

**Fig 3:**
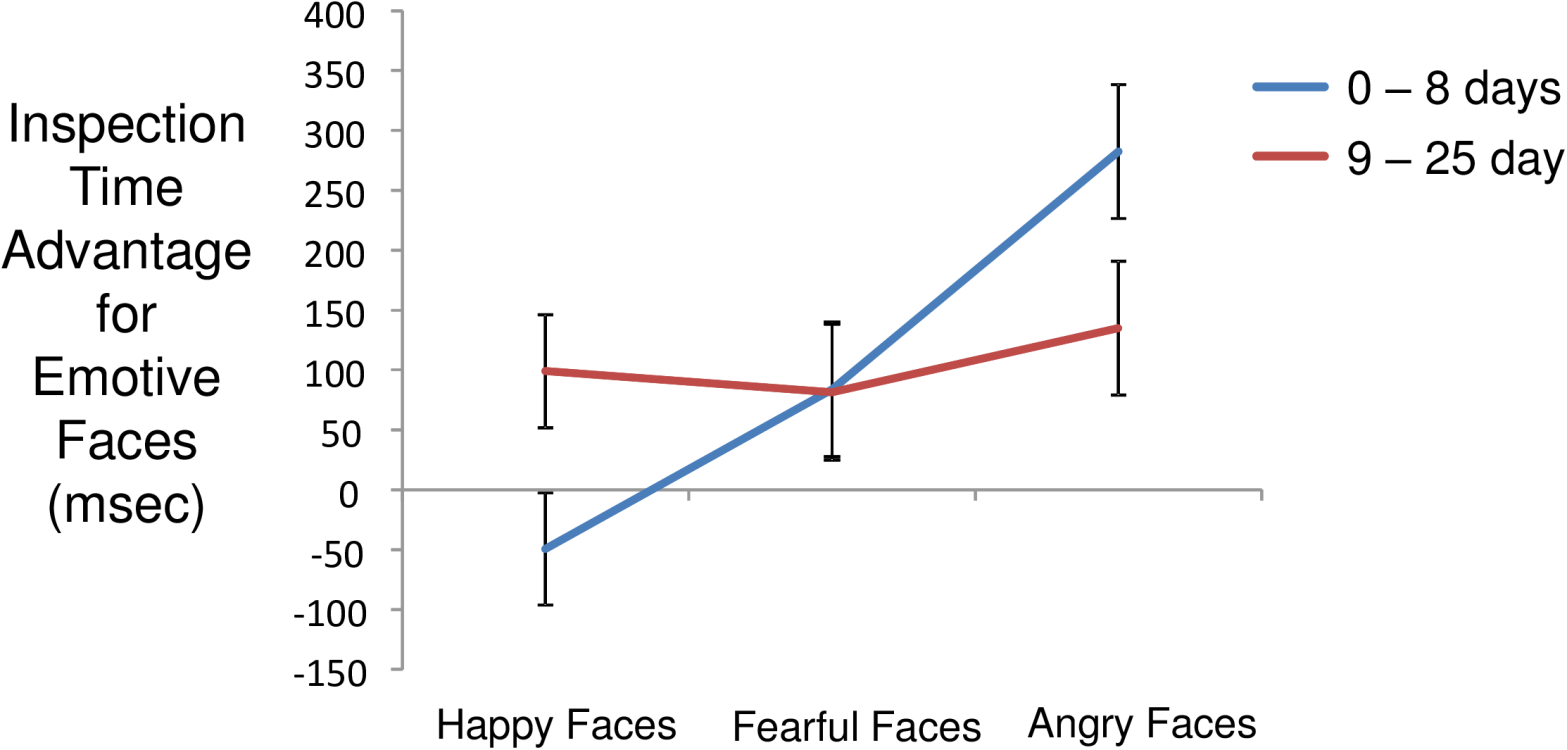
Interaction of contrast and accumulated days with canine (median split) on inspection time differences favoring emotive over neutral faces. Population marginal means derived from fitted model. Error bars are standard errors.

Mean inspection time for paired facial stimuli was 1853 msec (s.d. = 1245 msec). Within face pairs, mean inspection time for emotive faces was 976 msec (s.d. = 851 msec) and for neutral faces was 878 msec (s.d. = 807 msec). Per trial inspection time differences elicited by emotive versus neutral faces exhibited a mean of 98 msec (s.d. = 1096 msec), again indicating a bias in favor of emotive versus neutral stimuli. Modeling inspection time differences detected a main effect of emotion contrast (angry > fearful > happy; F(2,2206) = 12.9, p < 0.0001) and an interaction of contrast and canine presence (F(2,2206) = 4.37, p = 0.013). As shown in Fig 2, canine presence was associated with attenuation of bias towards angry faces. Decomposing this interaction, the effect of canine presence was significant for angry faces (t(73) = 2.27, p = 0.027), but not for fearful faces (t(73) = 0.49, p = 0.66), or for pleasant faces (t(73) = 0.65, p = 0.53). The effect of days post admission was not significant (F(1,12) = 0.03, p = 0.88). The model including days accumulated with canine detected a main effect of contrast (F(2,2206) = 12.9, p < 0.0001) and an interaction of contrast and days accumulated with canine (F(2,2206) = 7.1, p = 0.0008). As shown in Fig 3, longer periods of companionship were associated with modification of attentional bias towards happy faces and away from angry faces, with little change in attention to fearful faces. Only the increase in attention to happy faces was significant (F(1,74) = 5.74, p = 0.019; fearful: F(1,74) = 0.12, p = 0.73; angry: F(1,74) = 2.93, p = 0.091). Inspection time difference also exhibited an interaction of set and contrast (F(10,2205) = 5.47, p < 0.0001) indicating that the sets of facial image were not equivalent in inducing relative attentional bias across happy, fearful, and angry faces. This finding is not surprising in light of the fact that the facial stimulus sets were composed without the benefit of the valence ratings that were available for the IAPS images.

## Discussion

This study examined associations between attentional bias toward emotive image content and service canine presence/familiarity in a small but intensively-studied sample of U.S. military veterans with chronic severe PTSD. Measuring differential inspection times over paired images indexed visual attentional biases in a straightforward manner and yielded robust effects of attentional bias toward aversive content in line with prior studies [7–10]. Our findings suggest that the close physical presence of a service canine with which the participant had spent that day and a variable number of prior days and nights significantly attenuated bias toward generic aversive image content, implying modified appraisal of broad affective categories. On the other hand, the pattern of responses to facial stimuli suggested that the presence of a service canine specifically attenuated visual attention toward angry faces. More aggregated time spent with the service canine was also associated with modification of attentional bias toward emotive faces, specifically, increased time spent looking at pleasant facial expressions. As a result, the pattern of responses was to a degree orthogonal to the constructed distinction between generic and social tasks.

What light do these findings shed on attentional bias in PTSD and related disorders, particularly its modifiability? Studies have shown that the modifiability of attentional bias by a negative mood induction distinguishes social phobic children from control children [43], and excessive worriers from non-worriers [44, see also 45]. Though a standing attentional bias to threat in PTSD has been repeatedly confirmed [5], it is modifiability that underwrites utility as a treatment target. Though trauma-related stimuli have been used to "prime" various aspect of cognition in PTSD [e.g. 46], and a compelling within-task induction of attentional bias toward threat cues (a.k.a. "hypervigilance") has been demonstrated in normals [47], the comparative modifiability of attentional bias in PTSD has been little studied outside of the treatment literature. The current findings may be interpreted as supportive of attention bias modification-based approaches to PTSD; however, it is also possible that the presence of a familiar service canine brings additional neurobehavioral determinants to bear. Recent results suggest that human-canine interaction induces increases in circulating oxytocin [48], a "prosocial" hormone that modifies fear system function in persons with and without PTSD [49–56], and reduces attentional bias toward negative facial emotion [57–60]. If exogenous oxytocin can be shown to induce the attentional bias modifications observed here, the current results would be then be agnostic toward, rather than supportive of, the attentional bias modification training approaches now being trialed.

The advantage of angry over fearful faces in recruiting attention was unexpected in light of numerous studies demonstrating that fearful faces induce amygdala activation in controls [61–64] and excess amygdala activation in persons with PTSD [65, 66]; however, studies in adults employing both fearful and angry faces generally have not separated responses to these two classes. Though one small study in normal adults did find hemodynamic responses in the amygdala to be larger in response to fearful than angry faces [67, see also 68], greater attentional bias to angry than to fearful faces has been repeatedly demonstrated in young victims of maltreatment using multiple methodologies [69–73, see also 74]. Based on these findings, it might be proposed that contrasting responses to anger versus fear in adults with PTSD could illuminate the role of early trauma in the adult diagnosis.

This study has several limitations. The sample was composed exclusively of male U.S military veterans with chronic severe PTSD due to multiple trauma exposures. The findings may not generalize to females, or to persons with less severe PTSD subsequent to single-incident traumas. It is also noteworthy that the animals employed were bred to provide the highest levels of service. Breed quality may therefore have influenced the observation of effects of canine companionship. On “canine-plus” days participants spent nearly the entire twenty-four hours in close company with their canines. This "simulated-ownership" model will not have universal reach as an intervention. Situating the companion canine in the testing room always involved a three-way interaction between the participant, the canine, and an experimenter, leaving open the possibility that the canine-impacted two-way interaction between experimenter and participant may have independently influenced attentional bias. This possibility might be addressed by behavior analysis combined with oxytocin sampling [48, 75]. Future studies may also wish to include a psychometric assessment of the participant-canine bond [76].

### Conclusion

In this sample of veterans, canine companionship shifted attention to emotive visual stimuli in a broadly positive direction. Some support for specific adaptive effects on attention to human facial emotion was also obtained. These results lend support to the modifiability of motivated attention in combat Veterans with chronic severe PTSD. The comparability of such effects to those of the evidence-based psychotherapies for PTSD remains to be assessed.

## Supporting information

S1 File Supporting information. Table A. IAPS Image Pairs by Set.
Table B. NimStim Image Pairs by Set.
Fig A. Representative Scenes Task Stimulus. Representative scenes task stimulus presented with one participant's gaze tracks aggregated over all 64 trials in order to obscure the image in accordance with user agreement. Samples are summed per image and the difference between emotive and foil sums interpreted as attentional bias.
Fig B. Distribution of Attentional Bias by Task.
Fig C. Normal Probability Plots of Model Residuals.
(PPTX)
