# Supplementary material for: Canine companionship is associated with modification of attentional bias in posttraumatic stress disorder
Source: PLoS One. 2017 Oct 11;12(10):e0179912. doi: 10.1371/journal.pone.0179912 (PMC5636264; doi:10.1371/journal.pone.0179912)
Supplement: S1 File — Table A. IAPS Image Pairs by Set. Table B. NimStim Image Pairs by Set. Fig A. Representative Scenes Task Stimulus. Representative scenes task stimulus presented with one participant's gaze tracks aggregated over all 64 trials in order to obscure the image in accordance with user agreement. Samples are summed per image and the difference between emotive and foil sums interpreted as attentional bias. Fig B. Distribution of Attentional Bias by Task. Fig C. Normal Probability Plots of Model Residuals. (PPTX) [file pone.0179912.s001.pptx]

## Slide 1
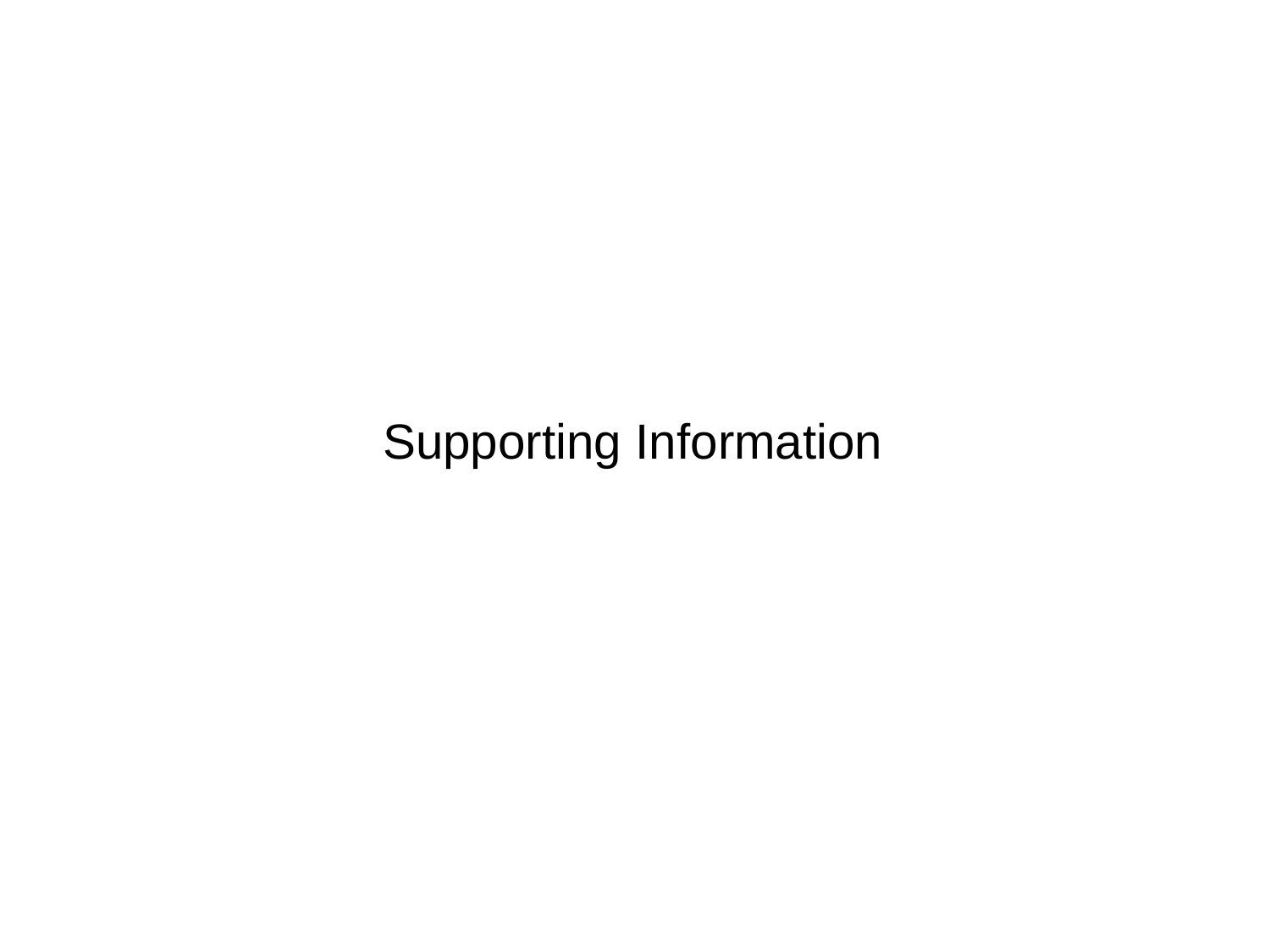

Supporting Information

## Slide 2
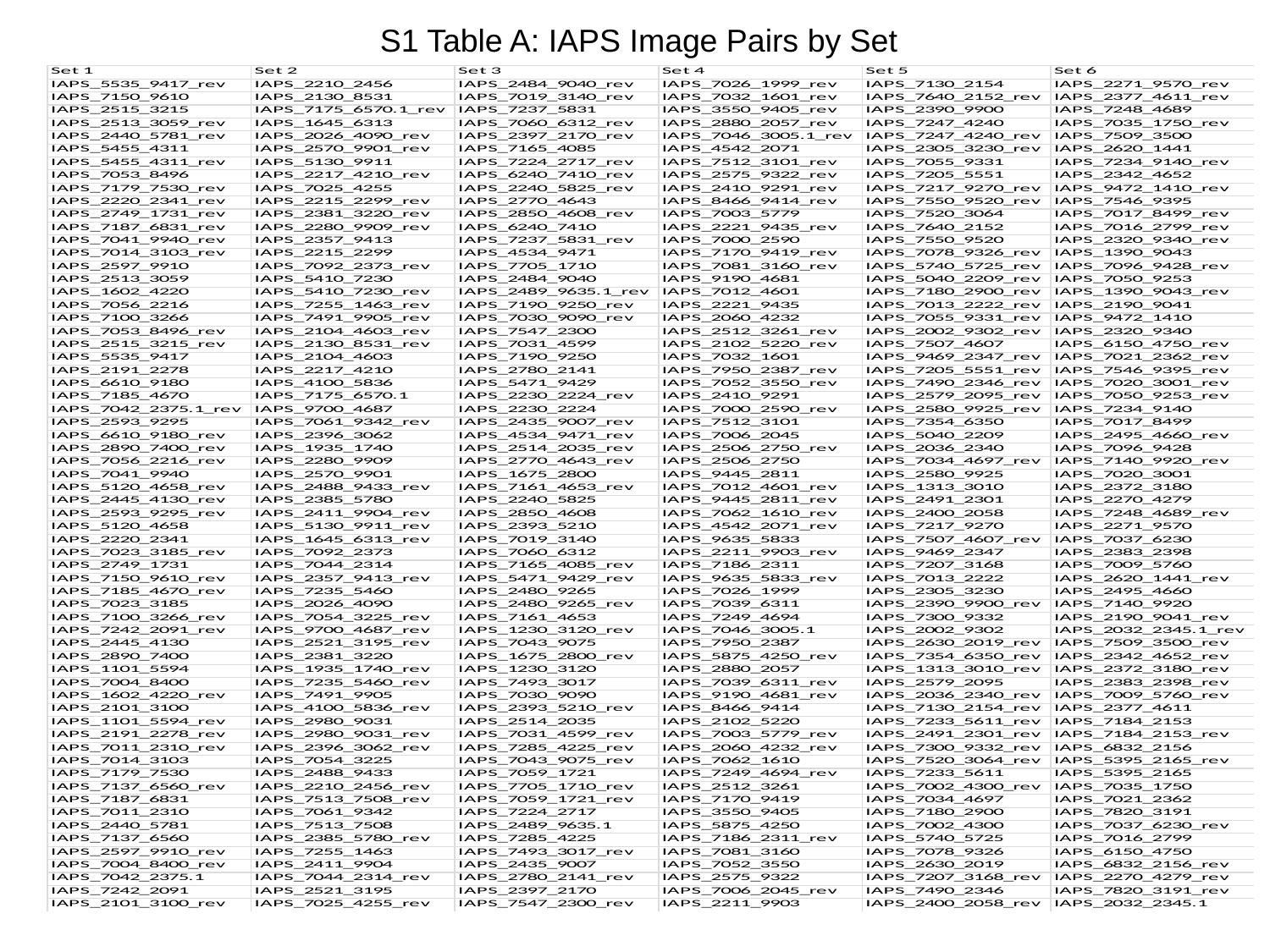

S1 Table A: IAPS Image Pairs by Set

## Slide 3
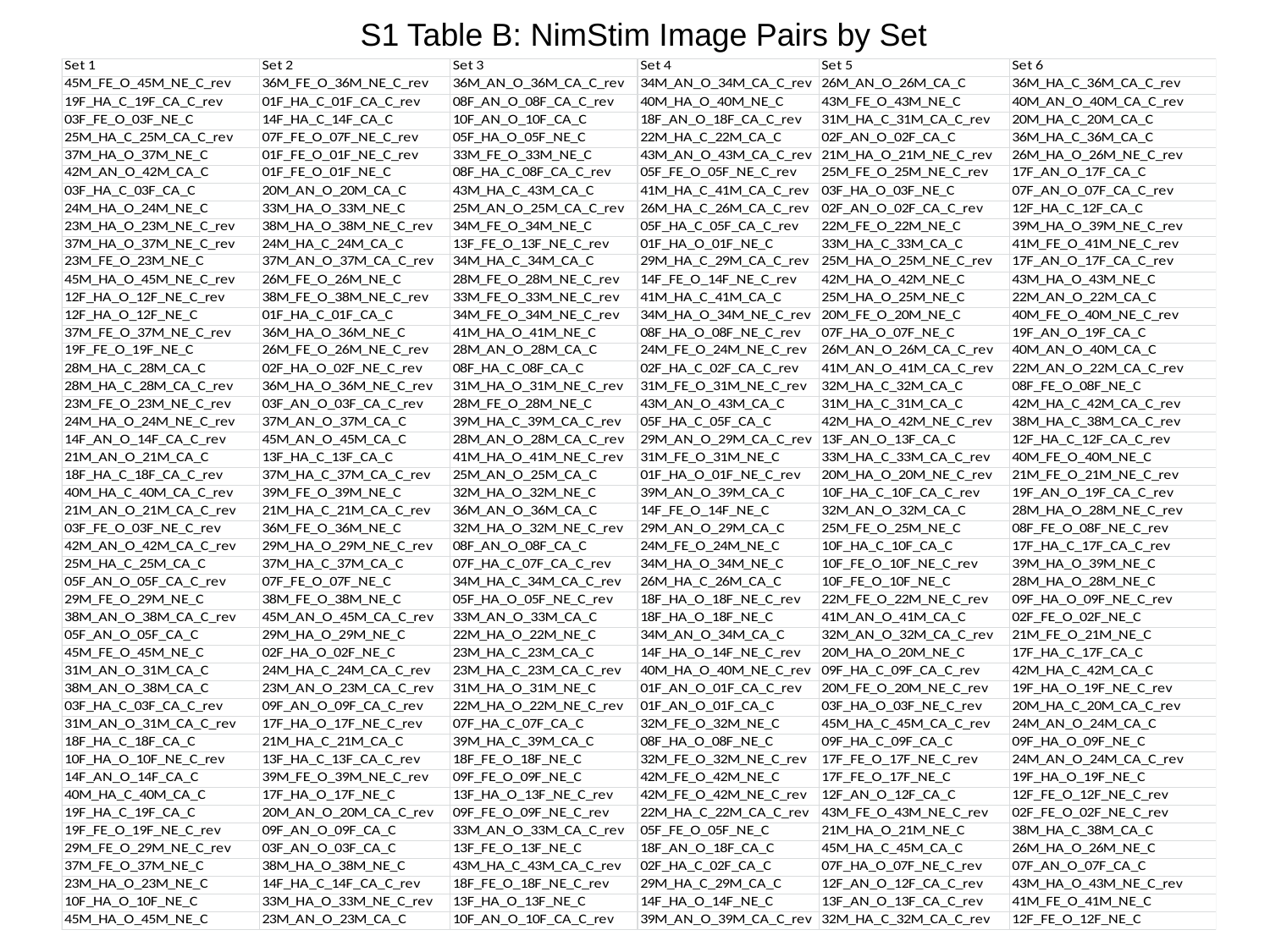

S1 Table B: NimStim Image Pairs by Set

## Slide 4
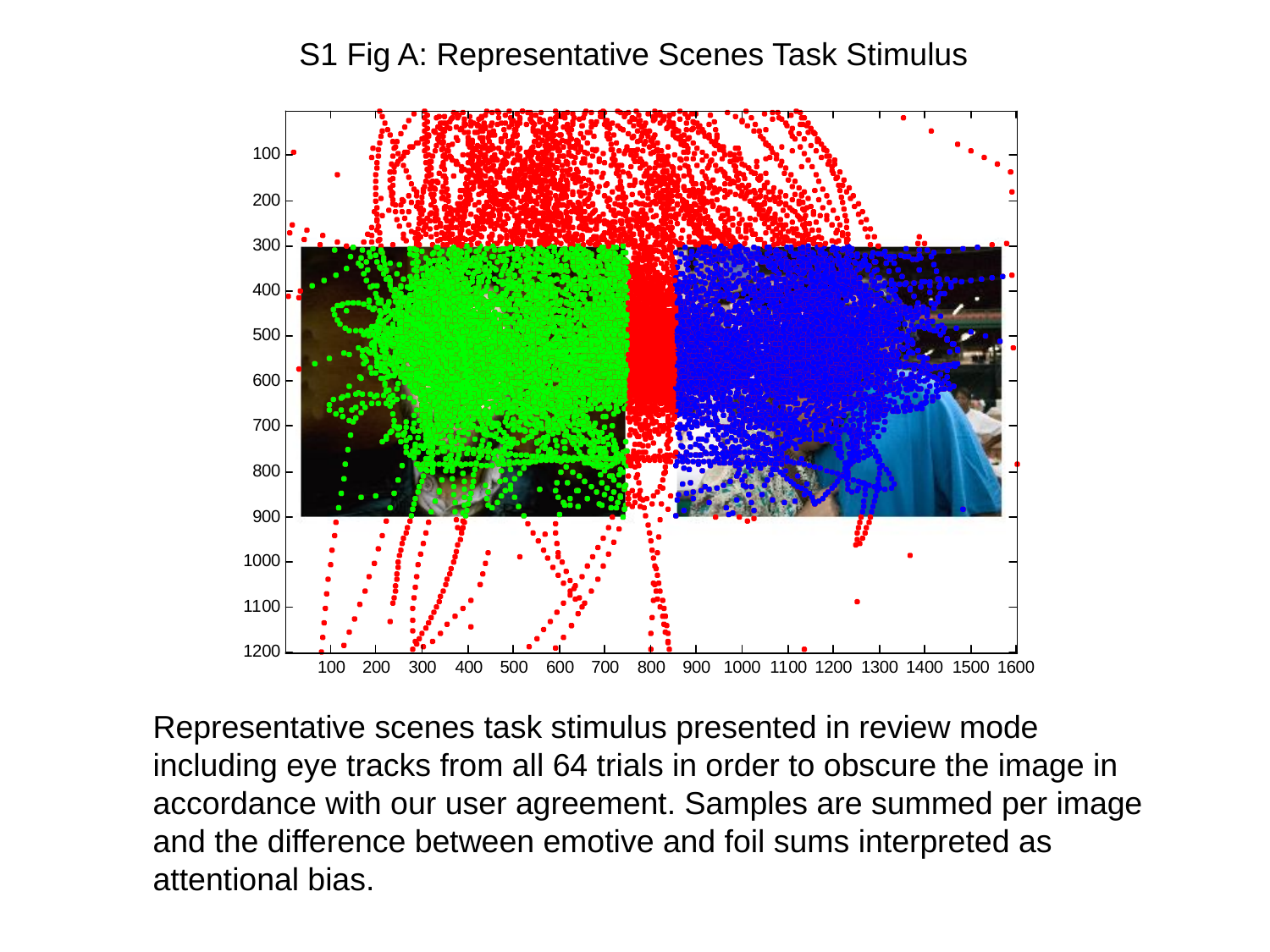

S1 Fig A: Representative Scenes Task Stimulus
Representative scenes task stimulus presented in review mode including eye tracks from all 64 trials in order to obscure the image in accordance with our user agreement. Samples are summed per image and the difference between emotive and foil sums interpreted as attentional bias.

## Slide 5
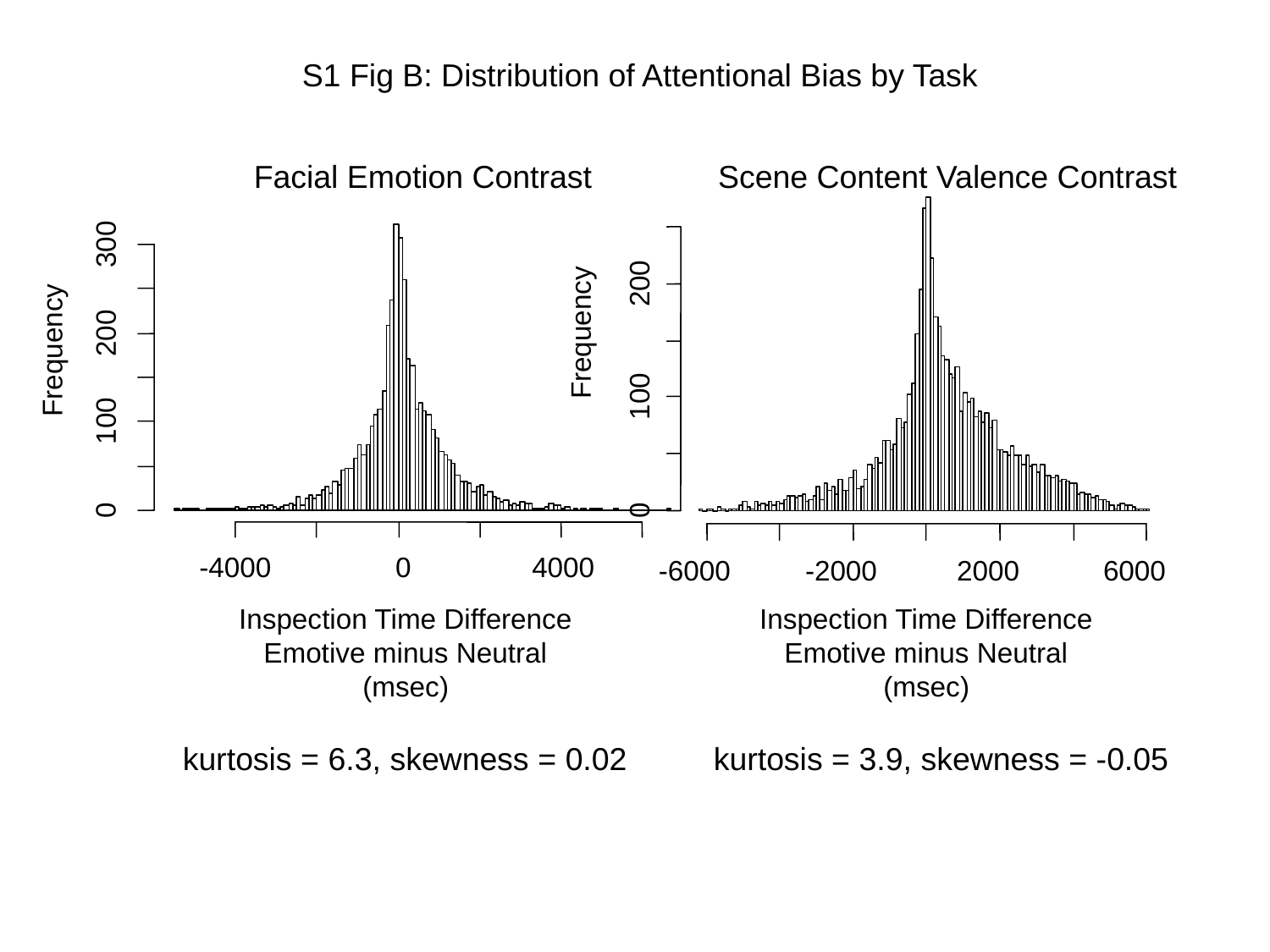

S1 Fig B: Distribution of Attentional Bias by Task
Facial Emotion Contrast
Scene Content Valence Contrast
200
Frequency
100
0
-6000
-2000
2000
6000
Inspection Time Difference
Emotive minus Neutral
(msec)
300
200
Frequency
100
0
-4000
0
4000
Inspection Time Difference
Emotive minus Neutral
(msec)
kurtosis = 6.3, skewness = 0.02
kurtosis = 3.9, skewness = -0.05

## Slide 6
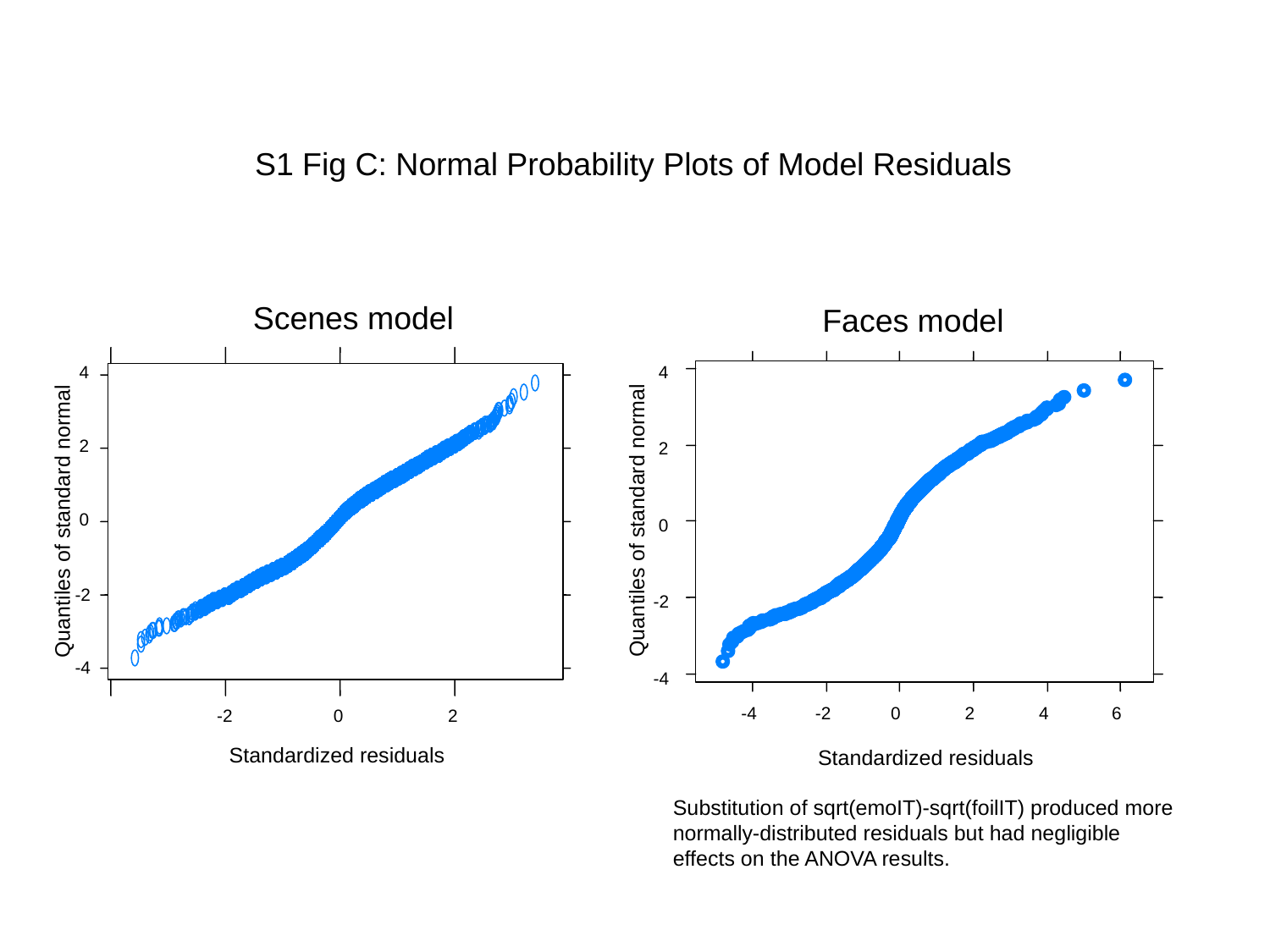

S1 Fig C: Normal Probability Plots of Model Residuals
Scenes model
Faces model
4
2
Quantiles of standard normal
0
-2
-4
-2
0
2
Standardized residuals
4
2
Quantiles of standard normal
0
-2
-4
-4
-2
0
2
4
6
Standardized residuals
Substitution of sqrt(emoIT)-sqrt(foilIT) produced more normally-distributed residuals but had negligible effects on the ANOVA results.
